# Characterization of a new mitophagy reporter uncovers requirement of BNIP3 for hypoxia-induced mitophagy in Drosophila

**DOI:** 10.21203/rs.3.rs-9407058/v1

**Published:** 2026-05-11

**Authors:** Hubert Osei Acheampong, Emily Rozich, Ryan Insolera

**Affiliations:** Wayne State University

**Keywords:** mitoSRAI, matrix-QC, autophagy, mitophagy reporters, mitophagy flux, Drosophila melanogaster, BNIP3, fluorescent proteins, lysosome, mitochondria

## Abstract

**Background.:**

Mitophagy is the cellular removal of unwanted mitochondria via the lysosome. Given the importance of this process to energy demanding tissues, mitophagy defects have been linked to various metabolic and neurodegenerative diseases. Mitophagy assessment tools are important for evaluating and quantifying mitophagy flux, which are useful in studying mitophagy pathways, mechanisms, and dysfunction. Mitophagy reporters are commonly used reagents to examine endpoint mitophagy flux. Following the generation of a new mitophagy reporter, mitoSRAI (mitochondrial Signal Retaining Autophagy Indicator), we introduced this reporter as a transgene into *Drosophila melanogaster* (Dm). We hypothesized that mitoSRAI will be capable of measuring mitophagic flux through microscopic visualization of the TOLLES:YPet fluorescence ratios, and bichemically through the relative persistence of TOLLES proteins in the lysosomes following YPet degradation.

**Results.:**

We found that when we express the mitoSRAI reporter in the Dm larval muscle wall and examine mitoSRAI flux by inducing mitophagy via hypoxia, we observe a significant increase in TOLLES only fluorescent signals and bands by confocal imaging and western blotting respectively. Complementarily, the readout of mitoSRAI is sensitive to conditions of mitophagy inhibition under hypoxia. To validate our results, we compared mitoSRAI to a similarly constructed reporter, matrix-QC, and found that mitoSRAI is less responsive to neuronal and fat body mitophagy flux manipulations.

**Conclusion.:**

Overall, our work characterizes the strengths and weaknesses of the application of the mitoSRAI reporter in Dm. We demonstrate with the mitoSRAI reporter that BNIP3 is an important mediator for hypoxia-induced mitophagy in Dm.

## Background

Mitophagy generally refers to the selective removal of damaged or excess mitochondria via an isolation membrane (mitophagosome) which is delivered to the lysosome for breakdown (1). This quality control process is significant for maintaining a healthy pool of proper functioning mitochondria, especially in long-lived postmitotic tissues such as neurons and muscles (2, 3). Although mitophagy is unique from canonical macroautophagy (herein after referred to as autophagy), it employs crucial autophagy mediators like autophagy related (ATG) ATG1, ATG5, and ATG8 for the initiation and completion of the phagophore (4-6), and essential autophagosome-lysosome fusion SNARE (Soluble NSF Attachment Protein Receptors) protein, syntaxin 17 (STX17) for lysosomal fusion, and degradation (7). There are two well described forms of mitophagy: ubiquitin-dependent, and ubiquitin-independent (receptor-mediated) mitophagy. The ubiquitin-dependent system requires an E3 ubiquitin ligase to activate and promote mitophagy (8). Mitochondrial substrates are labelled with polyubiquitin tags by an E3 ligase such as Parkin [in the conventional PINK1(PTEN-induced putative kinase 1)/Parkin pathway] (8), which recruits soluble autophagy receptors including p62/SQSTM1 (sequestosome 1), OPTN (optineurin)/ fly homolog Kenny (9, 10), and NDP52 (Nuclear Dot Protein 52 kDa) (11-13). These mediators initiate the engulfment of the mitochondria by the phagophore via LC3 (microtubule-associated protein 1A/1B-light chain 3) interaction to form the mitophagosome that is eventually degraded by the lysosome (1). In contrast, the receptor-mediated pathway utilizes resident OMM (Outer Mitochondrial Membrane) proteins like BNIP3 (BCL2 and adenovirus E1B 19-kDa-interacting protein 3), NIX/BNIP3L (Nip3-like protein X/BNIP3-like), and FUNDC1 (FUN14 domain-containing 1) that directly recruit, and interact with the phagophore LC3 to form the mitophagosome that gets degraded in the lysosome, typically in response to a hypoxic stimulus, and during cell or organismal development (14-17). Despite the well-defined outline of the mitophagy pathway, there are diverse documentations of the rate of mitochondrial turnover (mitophagy flux) that occurs in different tissues and organisms. Dysfunctions in mitophagy flux have been implicated in debilitating human conditions including neurodegenerative diseases, cardiovascular and metabolic disorders, and cancer (2, 3, 18, 19). Research into mitophagy flux and dynamics in various tissues in vivo is critical to future disease interrogations.

The mitophagy pathway has historically been studied in systems where in vitro cell cultures were exposed to toxic levels of chemical uncouplers, namely CCCP (carbonyl cyanide m-chlorophenyl hydrazone) and FCCP (carbonyl cyanide p-(trifluoromethoxy) phenylhydrazone) to cause mitochondrial membrane depolarization, damage, and induce mitophagy (8, 20-22). This approach was used in the discovery of the PINK1/Parkin-dependent mitophagy pathway (8, 21) which defined mitophagy as a stress response pathway. Notwithstanding the advancement in the field regarding the elucidation of key mitophagy pathways and their assessments in vitro, physiological measurement, tracking, and quantification of in vivo mitophagy was majorly difficult until the advent of fluorescent mitophagy reporters (FMRs). FMRs are generally pH-dependent and modify their emission wavelengths between the different pH in the mitochondria and the lysosome (23-25). Additionally, lysosomal enzymes digest the protease-sensitive component of FMRs (23, 25). These modifications afford the distinction between lysosomal and non-lysosomal FMRs, providing an index for mitophagy flux. Currently applied FMRs in order of recency include mitoKeima, mito-QC, matrix-QC, and mitoSRAI although others like mitoTimer, and dsRed have been documented in literature (23-30). mitoKeima consists of a single pH-dependent protein that targets the COXVIII (Cytochrome c Oxidase subunit 8) subunit of the mitochondrial matrix (24, 26, 29, 30), and was made for tracking mitophagy flux in live tissues making it incompatible with fixation methods (31). Because of this drawback, the fusion FMR composing of EGFP (Enhanced Green Fluorescent Protein) and mCherry fluorescent proteins, mitoQC, was generated, which was validated for use in fixed tissue (32, 33). The persistence of the mCherry in the lysosome is used as a proxy for an endpoint mitophagy readout. However, because of the OMM localization of mitoQC, it was susceptible to aberrant proteasomal degradation (34). To overcome this, matrix-QC, which localizes to the mitochondrial matrix was produced (33, 35).

In the quest of developing an FMR that was more specific, robust, and reliable, the mitoSRAI reporter was generated. One major limitation the mitoSRAI sought to address was the FRET (Förster resonance energy transfer) component of the fusion FMRs. The donor molecule of the FRET for the existing fusion reporters (mito-QC and matrix-QC) is the acid and protease-sensitive EGFP which translates into variable donor excitation of the mCherry hence causes inconsistent mitophagy readout (23). The mitoSRAI consists of the acid and protease-sensitive YPet, and acid and protease-resistant TOLLES. The TOLLES protein is the donor and YPet, the acceptor molecule in the mitoSRAI FRET (23), an improvement over the previous FMR FRET relationship. This, in theory, should improve the robustness, accuracy, and reliability of the mitoSRAI in reporting endpoint mitophagy. Moreover, to enhance the specificity of the reporter to the mitochondrial matrix (COXVIII), mitoSRAI has additional modifications at its c-terminus with degrons (CL1 and PEST) that signal proteasomal degradation of non-mitochondrial localization of mitoSRAI (23, 36).

Although the mitoSRAI has been validated in vitro (23), its in vivo characterization is lacking. Here, we provide robust in vivo validation of mitoSRAI using Dm as a model system utilizing different tissues. Dm is a useful organism for this study because of its tractable genetic system that allows for the generation and expression of transgenes, commonly with the GAL4-UAS system (37-39). Also, there is well-established documentation of the conservation of autophagy and mitophagy pathways between mammals and Dm which makes Dm convenient for studying various mitophagy-related human disease processes (40). Lastly, previously established FMRs have been well characterized using Dm (26, 41). We employ the late second instar (L2), early third instar (L3) larvae, and adult Dm for the various mitoSRAI mitophagy flux assessments. In comparison, we provide auxiliary testing of mitophagy flux readout of matrix-QC (chosen because of its analogy to mitoSRAI in structure and localization, and its amenability to fixation) under specific conditions in which we have applied the mitoSRAI. This work provides in vivo mitophagy-reporting characteristics of two different FMRs and their suitability for specific future mitophagy-related applications in Dm and potentially other models.

## Materials and Methods

### Drosophila husbandry, stocks, and procedures

Drosophila were reared under standard conditions at 25°C and in a 12h:12h light:dark cycle incubator. Fly stocks were maintained on a 10% yeast/sugar diet (regular food). All larval experiments utilized L3 under the above conditions. The following fly strains were obtained from Bloomington Stock Center: W^1118^ (BL #5905), D42-Gal4 (BL #8816), UAS-luciferase RNAi (Control RNAi) (BL #31603), UAS-Drp1 RNAi (BL #67160), UAS-Atg5 RNAi (BL #34899), UAS-matrix-QC (BL #602462), nSyb-Gal4 (BL #51635), Mef2-Gal4 (BL #50742), DA-Gal4 (BL #93111), UAS-Atg8a-mCherry (BL #37750), cgGal4 (BL #7011), and UAS-STX17 RNAi (BL #25896). Atg5^5cc5^ Dm line was a gift from (G Juhasz, Eötvös Loránd University, Hungary). Kenny^null^ mutant was made as previously described (9).

The mito-SRAI_pcDNA3 plasmid was obtained from RIKEN BRC. The full sequence of the mitoSRAI reporter was subsequently synthesized by Genscript Inc. (Piscataway, NJ) and sub-cloned into the pWalium10-moe vector with the EcoRI/NheI restriction sites. The completed vector (pWalium10-mitoSRAI) was then sent to BestGene, Inc (Chino Hills, CA) for injection and generation of transgenic flies. The UAS-mitoSRAI transgene was introduced into the genome by PhiC31-mediated recombinase mediated cassette exchange (RMCE) into the attP40 landing site on second chromosome and the VK27 landing site on third chromosome.

### Immunohistochemistry

Preparation of samples expressing mitoSRAI and matrix-QC were adapted from previously published procedures (42). L3 larvae were selected based on visual and fluorescent markers where applicable and filet dissections as previously described (43) were performed in ice cold PBS. L3 fat body dissection was performed as described elsewhere (9, 44). The samples were immediately fixed in a neutral pH fixative (4% formaldehyde in 200mM HEPES, pH 7.0) for 20–30 mins at room temperature (RT). Samples were washed in PBS after fixation, incubated for 15 mins with 70% glycerol, and mounted using VectaShield mounting media (Vectorlabs). Samples were typically imaged within a day after mounting. Samples for experiments that involved co-staining with antibodies were prepared as above with the following additional components: Samples were washed 3 times in PBS at 10 mins intervals on a shaker then washed with PBST (PBS with 0.1% Triton X-100) 3 times for 5 mins per wash. The samples were then blocked for at least 30 mins in a blocking buffer (PBS-T supplemented with 5% normal goat serum). The samples were incubated overnight in primary antibody solution diluted in the blocking buffer overnight at 4°C. Samples were washed 3 times with PBST and then incubated in secondary antibody solution diluted in the blocking buffer for 2 hours at RT [10 μL of DAPI (4’,6-diamidino-2-phenylindole) was used as counter stain where applicable] followed by PBST washes. The samples were then mounted in VectaShield media ready for confocal imaging. Only male larvae were selected for the Atg5^5cc5^ experiments because this gene is on X chromosome.

### Confocal imaging

All imaging was done with a spinning disk confocal microscope equipped with a 63x oil immersion objective lens (NA 1.4), with Z step size between 0.2–0.3μm. The specifications of the microscope are a Leica DMI6000B body with an X-Light V2 spinning disk (Crest Optics), 16-bit CMOS camera (Photometrics), and LDI 7 laser launch (405, 445, 470, 520, 528, 555, and 640 nm) (Cairn Research and 89 north). *mitoSRAI imaging*: The YPet protein was imaged exciting a 520 nm laser and using a 540–580 nm emission filter. The TOLLES protein was imaged exciting a 445 nm laser and using a 460–500 nm emission filter. *matrix-QC imaging*: the EGFP protein was excited with a 470 nm laser and using a 485–535 nm emission filter. The mCherry protein excited with a 555 nm laser and using a 575–625 nm emission filter.

### Confocal image analysis

All image quantifications were performed with ImageJ. The mitophagy index of all the L3 body wall muscles was ascertained with semiautomated mQC counter plugin for ImageJ as previously published (45). Individual Z planes of acquired muscle images were closely scrutinized to determine the most representative Z plane, and the region of interest (ROI) was selected. The selected Z plane was Z-projected with maximum intensity. A few planes above and below the selected Z plane (15–20 planes) were included in the maximum projection of the 3D image into a 2D image. The number of mitolysosomes was counted with the mQC counter while optimizing the parameters of the counter for each image as previously described (45, 46). Muscle 12 of the L3 larval body wall muscles (47) was consistently imaged in all our L3 muscle imaging to ensure standardized comparisons. A range of 3–7 images of each sample were processed, and values averaged to a single data point. The L3 fat body mitophagy index in the matrix-QC experiment was generated through the above process. L3 VNC (ventral nerve cord) matrix-QC images were processed by maximally projecting 5–7 Z planes. The number of mid-line motor neuronal cell bodies in the single 2D image was counted as well as the number of mitolysosomes. For the *Drp1 (Dynamin-related protein 1)* RNAi samples, motor neuronal cell bodies containing hyperfused or enlarged mitochondria indicative of *Drp1* KD (knock down) were counted. A range of 2–3 VNC images were assessed for each sample and the total number of cell bodies (**a**) and mitolysosomes (**b**) recorded. The number of mitolysosomes per cell defined as “mitophagy index” was calculated as the ratio of **b** to **a**. The relative mitophagy index was calculated as the ratio of the mitophagy index in the manipulated condition to the average mitophagy index in the control condition. An average of 40 cell bodies were assessed for each sample.

### Biochemical assessment (Western blotting)

For all L3 body wall muscle lysates, L3 larvae were dissected with all extraneous organs and tissues removed leaving only the L3 body walls. Each dissection was performed typically between 3–5 mins, and samples were put in a 1.5mL microcentrifuge tube containing ice cold PBS kept on ice. Excess PBS was removed after all dissections were completed. Each tube containing 5–6 dissected L3 larvae was placed in a −80°C freezer for 10 mins. Lysis buffer warmed at 95°C was used for each sample. Each sample was homogenized in lysis buffer with pestle, sonicated for 15s at 50% amplitude on ice and heated at 95°C for 10 mins. Samples were centrifuged at maximum speed (13.3×1000/min) for 10 mins at RT. Supernatant protein samples were obtained which were used for subsequent SDS-PAGE gel electrophoresis. 6μL of sample was loaded in each well of a 4–20% gradient precast gel (Mini-Protein TGX Gels with maximum capacity of 15μL). Gel was run at 160 V for 10 mins initially and 180 V for an additional 20–25 mins. Trans-Blot Turbo system was used to transfer the proteins onto PVDF membranes. Membranes were washed in 10 mL of 1% TBST. Membranes were blocked with 5% non-fat dry milk prepared in TBST. Primary antibody solution made in 2% milk (with 0.05% sodium azide) was used to incubate membranes overnight at 4°C. PVDF membranes were washed in 1% TBST 3 times at 10 mins intervals and incubated in 1:5000 secondary antibody solution (in 2% milk in TBST) for 1 hour. Membranes were washed 3 times at 10 mins intervals in TBST and developed with (SuperSignal^™^ West Pico Plus) chemiluminescent substrate before imaging with the Azure Biosystems 300 for blots and gels. Adult fly head lysates were prepared by collecting 15–20 heads directly into tubes containing 100 μL of preheated lysis buffer placed on ice. Tubes were frozen at −80°C for 10 mins and subsequently thawed and homogenized with pestle. The remaining steps are described as above with all incubation done on a shaker.

Lysis buffer (1x) is prepared from 2x homogenization buffer (20% SDS, 1.5mg/mL bromophenol blue, 10% glycerol, and 1M Tris-HCl at pH 6.8), 1M dithiothreitol (DTT) and 60mL milliQ water.

Total protein staining was done using the Direct Blue total protein staining protocol. Briefly, 800 μL of Direct Blue dye was mixed with 10 mL of Direct Blue solvent (40% ethanol + 10% acetic acid dissolved in milliQ water). The membrane blot was stained with the resultant solution for 10 mins, rinsed in the direct blue solvent for at least 10 mins and then with milliQ water. The membrane was air dried and imaged with the Azure Biosystems 300 for blots and gels.

### Biochemical analysis

Mitophagy flux analysis involved estimating band intensities using ImageJ plugin tool for gels. Band intensity peaks and area were generated utilizing the densitometry method. Band intensity areas were obtained for the “Intact mitoSRAI (**a**)” and the “Tolles only (**b**)” molecular weight bands. “Mitophagy flux” defined as TOLLES only band intensity per total specific anti-TOLLES protein recognition (“Intact mitoSRAI” and “TOLLES only” band intensities) was calculated as the ratio of **b** to (**a + b**) expressed as a percentage, with the expression, “(**a + b**)” serving as an internal control.

### Toxin treatments

#### Chloroquine treatment

2.5mg/mL of chloroquine diphosphate salt solution (48) was prepared in 15mL of milliQ water. 1ml of this solution was mixed with regular fly food on which L3 larvae were reared during hypoxia exposure.

##### FCCP treatment:

Early L3 larvae were fed with a 500μM (1:200 dilution of 100mM) dose of FCCP (dissolved in DMSO) mixed with 1mL milliQ water and 0.4g of instant fly food (Carolina Biological Supply, Formula 4–24). Duration of feeding is indicated in figure legends and in main text. Control conditions had only DMSO at the same concentration in food.

### Hypoxia, Starvation, and Feeding

For hypoxia exposure, L3 larvae were reared on regular food and kept in a (ProOx Model P110 BioSpherix) hypoxia chamber at 4% O_2_ for 24 hours. During starvation treatment, at least 15 late L2 or early L3 larvae were put on 20% sucrose (w/v) in PBS on a 35mm diameter petri dish for 3.5-4 hours. In feeding conditions, 0.4g of instant fly food was mixed with 1 mL milliQ water in a 35mm diameter petri dish and at least 15 larvae were kept on this food for 3.5-4 hours. All the samples for these conditions were kept in a fly incubator at 25°C. The L3 fat body starvation experiments involved incubating dissected larvae for 5–8 minutes in 1 mL of 1:100 lysotracker red dye diluted in PBS before fixation. The subsequent steps of immunohistochemistry are as described earlier.

### Antibodies

PDH (goat anti mouse PDHA1, 1:500, ab110334, Abcam), α-TOLLES (anti rabbit monomeric AzamiGreen (mAG), 1:500 for both immunohistochemistry and western blotting, PM052M, MBL International), and α-Tubulin (anti mouse tubulin, 1:1000, E7-s, DHSB). All secondary antibodies for western blotting were prepared at 1:5000 dilution.

### Drosophila cell culture, transfection and western blotting

S2R+ cells (DGRC Stock 150; https://dgrc.bio.indiana.edu//stock/150; RRID:CVCL_Z831) were maintained at 25°C in complete M3 (Sigma, S8398) BPYE media supplemented with 10% heat-inactivated fetal bovine serum (FBS). Subconfluent (70–80%) cells were seeded at 2x10^6^ cells per 2mL of complete media (M3 + BPYE + 10%FBS) for transfection. Cells were separately transfected with specific plasmids containing the UAS constructs of either YPet, TOLLES or mitoSRAI (YPet +TOLLES), and the empty vector (EV), pWalium10-moe vector, using the FuGENE HD transfection reagent (Promega, E231A). All these groups of cells were co-transfected with pMT-Gal4 whose expression was driven by the addition of CuSO_4_ (copper II sulfate) to the media at a final concentration of 0.5mM. Successful transfection of the fluorescent proteins was confirmed by visualizing the various cell transfection groups under a fluorescence microscope (leica-microsystems, Model; MSV269) using the GFP LED filter to detect green fluorescence. S2R+ cells were washed with ice-cold PBS 48 hours after transfection and lysed with a mixture of RIPA buffer (thermo scientific, 89900) and protease inhibitor cocktail (Thermo Scientific, 1861281). Extracted protein concentrations were estimated from the Bradford assay (Bradford reagent, thermo scientific, 1863028). 2x Laemmli sample buffer (BIO-RAD, 1610734) was used for denaturing proteins in a water bath at 95°C for 5 mins and then centrifuged (at 16000 x g) for 1 min. Equal volumes of 20 μg of protein samples was loaded in each well for gel electrophoresis. Subsequent western blotting procedure is as described earlier.

### Statistics and software

Sample sizes were chosen based on previously published results. Data were analyzed using the unpaired two-tailed t-test (to directly compare two experimental groups), and one-way ANOVA with Tukey’s (Fig. 7B and 7D)/Dunnett’s (Fig. 4B) multiple comparisons test as applicable (to compare more than two groups) with the assumption of a normal distribution in all conditions tested. The number of biological replicates is indicated in each figure legend. All error bars represent mean ± SEM. A statistical comparison was considered significant when p < 0.05. All sample data obtained were analyzed and included in the quantifications except in Fig. 5D where iterative Grubbs test was used to omit outliers at p < 0.05. All analyses were performed with knowledge of the conditions examined. All graphical and statistical analyses were performed with GraphPad Prism version 9.3.1. Schematic figures were generated using BioRender. Final figure files were processed and compiled using the Adobe Creative Cloud suite software (Photoshop v27.5).

## Results

### mitoSRAI is specific and localizes to the mitochondrial matrix

**Figure 1:**
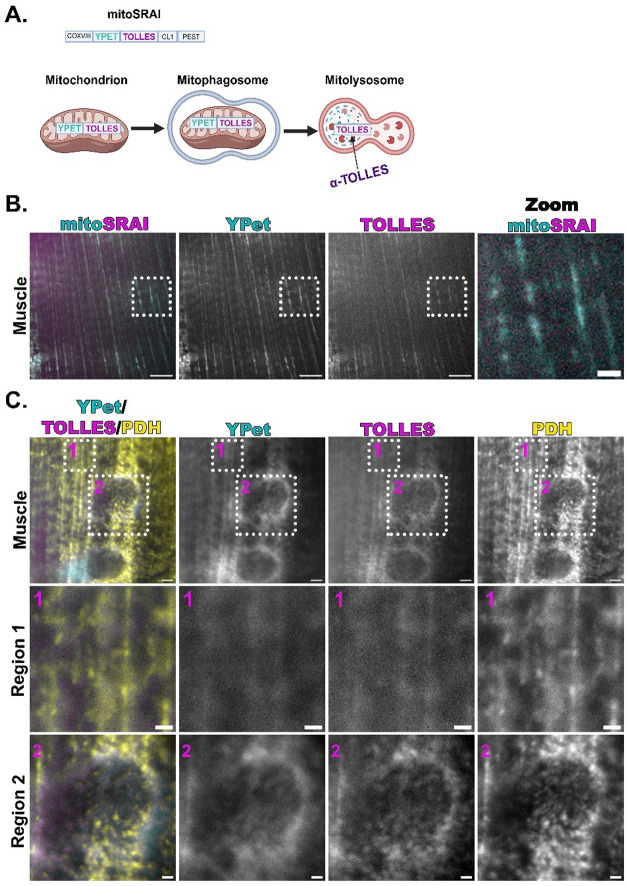
mitoSRAI reporter localizes to the mitochondria. **A.** Schematic of mitoSRAI reporter and its dynamics via the mitophagy pathway. **B** and **C** are representative images of L3 body wall muscles expressing mitoSRAI via the Mef2-Gal4 driver. **B.**YPet and TOLLES colocalizing on distinct structures. Scale bars, 10μm and 2μm (zoom image). **C.** YPet and TOLLES colocalizing with immunostaining for the mitochondrial matrix protein, PDH. Zoom images designated **1** and **2**represent intermyofibrillar and subsarcolemmal mitochondria respectively. Scale bars, 5μm, 2μm (for zoom image **1**) and 3μm (for zoom image **2**).

The basic composition of the mitoSRAI reporter and its turnover in the mitophagy pathway is shown in the schematic diagram in Fig. 1A. We first wanted to confirm the mitochondrial localization of the mitoSRAI. We expressed mitoSRAI in the L3 muscle (via Mef2-Gal4 driver) to determine if the YPet and the TOLLES proteins were localized to the same and distinct structures. We observed colocalization of these proteins to the same regions (Fig. 1B). To ascertain mitochondrial matrix localization, we immunostained for the endogenous mitochondrial matrix protein, PDH (pyruvate dehydrogenase), in samples where we have expressed the reporter. The PDH colocalized with the mitoSRAI proteins in these samples (Fig. 1C). Further, we show different regions of colocalization based on mitochondria pattern. Region **1** in **Figure C** shows dispersed mitochondria that are present in the myofibrils, while region **2** shows the reticular pattern of mitochondria in the subsarcolemmal region near the nucleus of the muscle.

### Monomeric AzamiGreen antibody (α-TOLLES) specifically recognizes mitoSRAI via TOLLES detection

**Figure 2:**
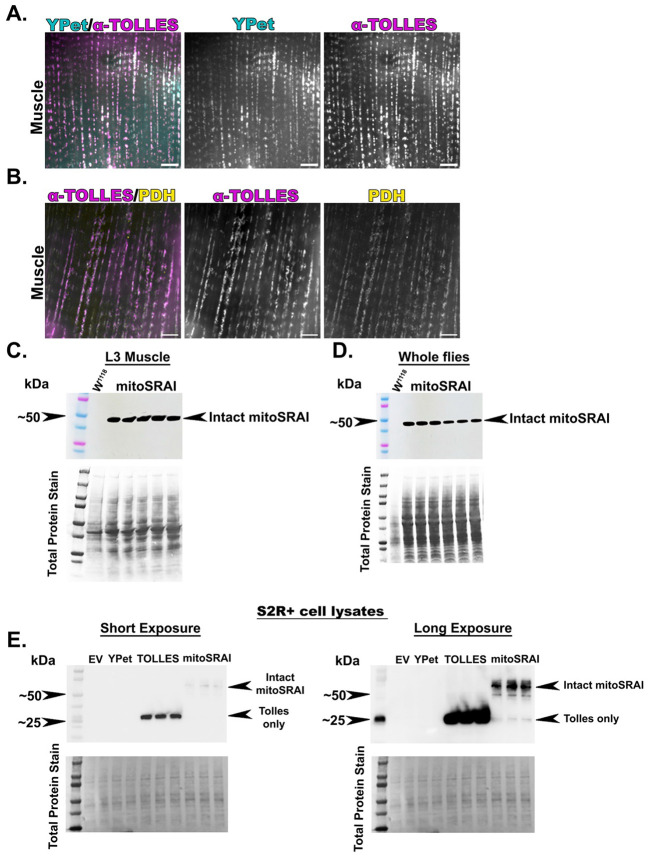
α-TOLLES antibody specifically recognizes TOLLES and mitoSRAI. **A** and **B** are representative images of L3 body wall muscles expressing mitoSRAI via the Mef2-Gal4 driver. **A.** α-TOLLES staining colocalizes with YPet fluorescence. Scale bar, 10μm. **B**. α-TOLLES staining colocalizes with immunostaining for the mitochondrial protein, PDH. Scale bar, 10μm. **C, D.** Western blot image showing the specificity of α-TOLLES antibody for mitoSRAI in both L3 muscles (C) and whole flies (D). Samples are protein lysates from larval body wall muscles (C) and whole flies (D) all expressing the mitoSRAI reporter with a muscle-specific Gal4 (Mef2-Gal4) in (C) and ubiquitously expressed Gal4 (Da-Gal4) in (D). W^1118^ (negative control) lane throughout this manuscript represents Dm protein lysates with no mitoSRAI expression. **E.** Samples are protein lysates from S2R+ cells with the individually expressed plasmids (via Cu^2+^ regulated pMT-Gal4) indicated in the figure. EV denotes cell lysates from cells transfected with empty vector and pMT-Gal4. Individual lanes represent separate sample protein lysates.

An antibody raised against monomeric AzamiGreen, hereafter referred to as α-TOLLES, has been previously shown to recognize TOLLES (23, 49), since TOLLES is derived from AzamiGreen. To test the specificity of α-TOLLES in immunostaining, we stained for this antibody in L3 muscle expressing mitoSRAI. Like in Fig. 1B, YPet (visualized by endogenous fluorescence) and α-TOLLES staining colocalized (Fig. 2A). We also observed α-TOLLES and PDH in similar regions when stained together, emphasizing its strict mitochondrial localization (Fig. 2B). We subsequently tested α-TOLLES’s capacity to probe mitoSRAI biochemically. Our western blotting results from L3 muscle-specific and whole-body expression (via DA-Gal4 driver) of mitoSRAI portrayed that α-TOLLES could detect “intact mitoSRAI” bands at the predicted size of the fusion of YPet and TOLLES (~ 50 kDa) (Fig. 2C and 2D). To verify that this α-TOLLES antibody exclusively recognizes TOLLES, we expressed YPet or TOLLES alone in cultured Drosophila S2R+ cells, collected cell lysates, and probed with the α-TOLLES antibody. The results demonstrated TOLLES migrates at ~ 25 kDa, and that the α-TOLLES antibody only recognizes TOLLES, and not YPet (Fig. 2E). Also, we probed for mitoSRAI with α-TOLLES in S2R+ cells where we have expressed the mitoSRAI reporter. We observed mitoSRAI band at the predicted size (~ 50 kDa) upon immunoblotting (Fig. 2E). These data (Figs. 1 and 2) provide evidence for the mitochondrial targeting specificity of the mitoSRAI reporter and that α-TOLLES selectively recognizes the TOLLES component of the mitoSRAI.

### mitoSRAI is sensitive to established conditions of mitophagy induction and inhibition.

**Figure 3:**
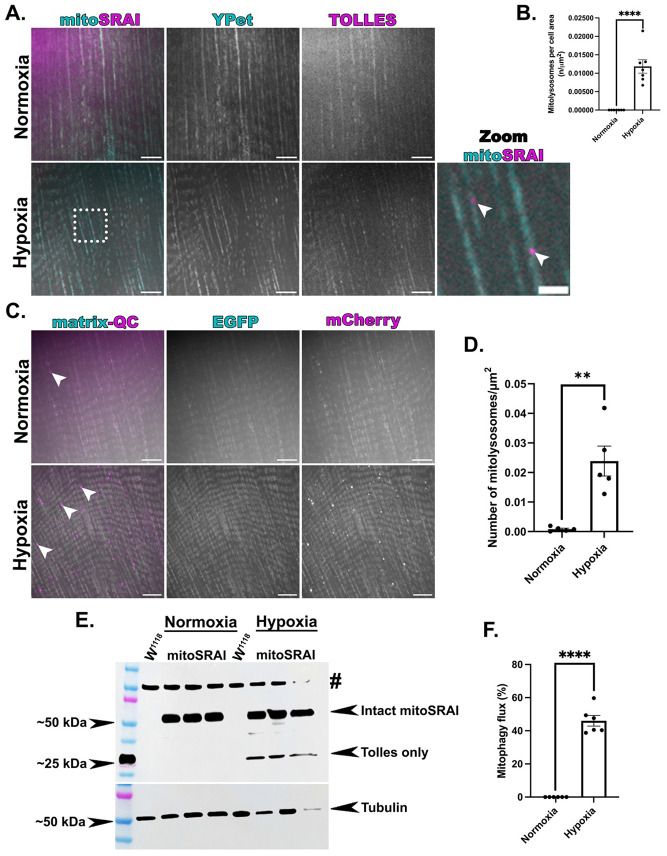
mitoSRAI and matrix-QC report hypoxia-induced mitophagy. **A.** Representative images of L3 body wall muscles expressing mitoSRAI via the Mef2-Gal4 driver. White arrow heads in zoom image pointing to magenta structures indicate examples of mitolysosomes. Scale bars 10μm, and 2μm (for zoom image). **B.** Quantification of mitophagy index from conditions in A where ****p<0.0001. Bars represent mean, with error bars representing SEM. Individual points represent one larva with n=7 larvae in each condition. **C.** Representative images of L3 body wall muscles expressing matrix-QC via the Mef2-Gal4 driver from animals exposed to hypoxia. White arrow heads show examples of mitolysosomes. Scale bar, 5μm. **D**. Quantification of mitophagy index from conditions in C. Bars represent the mean, with error bars representing SEM. Individual points represent one larva with n=5 larvae in each condition, **p<0.01. **E.** Western blot image showing protein lysates obtained from the larval body wall muscles expressing mitoSRAI via the Mef2-Gal4 driver. α-TOLLES antibody was used to probe for intact mitoSRAI and TOLLES only bands in each sample. **F.** Quantification of mitophagy flux in C where ****p<0.0001. Each lane represents an independent sample of 5-6 larvae protein lysates (n); n=6 independent samples were analyzed for each condition. # indicates nonspecific bands.

To demonstrate that mitoSRAI can report mitochondrial turnover, we relied on a robust mitophagy-inducing condition, hypoxia, that has been used to validate previous reporters in vitro and in vivo (26, 30, 50). We expressed mitoSRAI in L2/L3 muscle and exposed the larvae to 24 hours hypoxia in the treated group and normoxia in the control group. We observed increased “TOLLES only” signals in the hypoxic group (Fig. 3A) which was significantly higher than in the control group (Fig. 3B). We interpret these TOLLES only signals to be mitolyosomes, based on previous interpretation of other FMRs (32, 33, 51). To confirm our observation, we explored the dynamics of the established matrix-QC reporter in larval muscle under hypoxia, and we found an apparent increase in mitolysosomes (Fig. 3C) that was significantly higher than in the normoxia group (Fig. 3D). The consistent results in hypoxic conditions of matrix-QC and mitoSRAI expressed in larval muscle validate their specific readouts of mitophagic flux.

Biochemically, we tested the readout of mitophagy by mitoSRAI using the α-TOLLES antibody. We reasoned that bands less than ~ 50 kDa recognized by the α-TOLLES antibody may represent a degradation byproduct of the reporter containing the TOLLES protein, which can be used as proxy for end-stage mitophagy when detected with fluorescent microscopy *in situ* and via western blotting. While we observed intact mitoSRAI (~ 50 kDa) in both normoxic and hypoxic conditions, we saw a prominent second band at approximately 25 kDa in the hypoxia group only (Fig. 3E) which was significantly different from the control (Fig. 3F). This band is consistent with the “TOLLES only” portion (confirmed in Fig. 2E) of the mitoSRAI reporter in which the YPet protein has been degraded in the lysosome.

**Figure 4:**
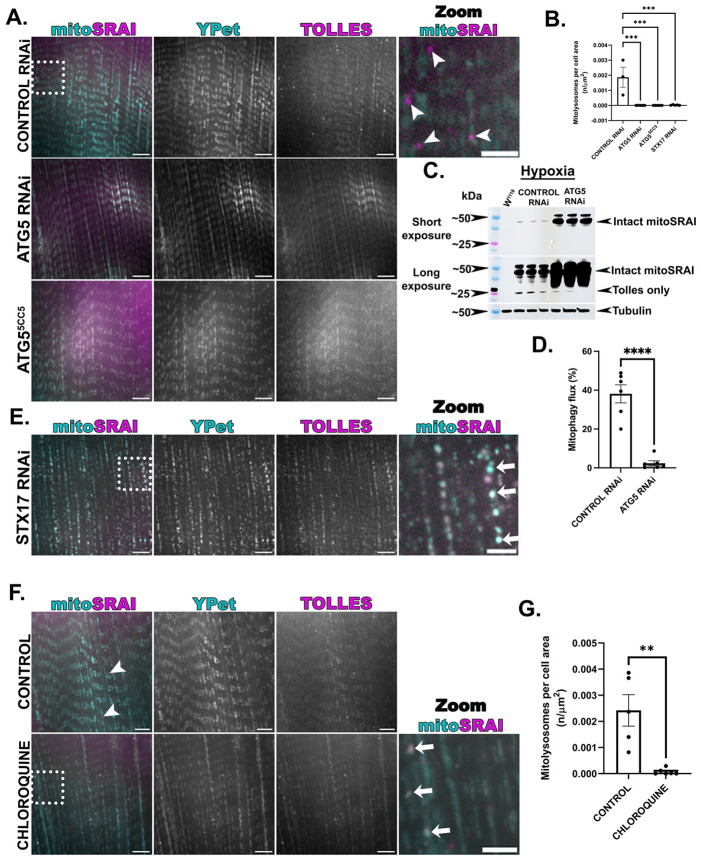
mitoSRAI flux readout is reduced in conditions of compromised autophagy. **A**,**E** and **F** are representative images of L3 body wall muscles expressing mitoSRAI via the Mef2-Gal4 driver. **A.** Genetic knockdown and ablation of the core autophagy gene (*Atg5*). Zoom image with white arrow heads pointing to magenta (TOLLES only) structures indicate examples of mitolysosomes. Scale bars 10μm, 2μm (zoom image). **B.** Quantification of mitophagy index in the genetic knockdown conditions in A, ***p<0.001. Bars represent mean, with error bars representing SEM. Individual points represent one larva with n=3 larvae in control RNAi (luciferase RNAi represents control RNAi throughout this article), and n=5 larvae in the remaining conditions. **C.** Western blot image showing protein lysates obtained from the larval body wall muscles co-expressing mitoSRAI via the Mef2-Gal4 driver, along with the RNAis indicated above. α-TOLLES antibody was used to probe for intact mitoSRAI and TOLLES only bands in each sample. **D.** Mitophagy flux quantification of **C**, ****p<0.0001. Bars represent mean, with error bars representing SEM. Each lane represents an independent sample of 5-6 larvae.; n=6 independent samples were analyzed for each condition. **E.** *Stx17* KD in larval muscles in animals exposed to hypoxia. Zoom image with white arrows pointing to accumulating “mitophagosomal structures”. Scale bars 10μm, 2μm (for zoom image). **F.** Pharmacologic inhibition of bulk autophagy via chloroquine (1mL of 2.5mg/mL concentration). White arrow heads indicate examples of mitolysosomes. White arrows in zoom image show examples of accumulating “mitophagosomes”. **G.** Mitophagy index quantifications of conditions in F, **p<0.01. Bars represent mean, with error bars representing SEM. Individual points represent one larva with n=5 larvae in control, and n=6 larvae in chloroquine-treated group. Scale bars 10μm, and 2μm (for zoom image).

To establish the reportage of mitophagy events by mitoSRAI, we tested if it was responsive to different known mitophagy-inhibiting scenarios involving genetic and pharmacologic manipulations. Downstream autophagy genes including *Atg5* and *Stx17* have been shown to be crucial to process and complete autophagy irrespective of the type of autophagy (7). Consistent with this paradigm, we observed a significant decrease in the mitophagy readout under hypoxia when we knocked down *Atg5* in L2/L3 muscle (Fig. 4A and 4B). We verified the reduction in the mitophagy readout in the *Atg5* knockdown condition biochemically where we observed noticeable reduction in the “TOLLES only” band intensity (Fig. 4C) which reflected quantitatively in the mitophagy flux assessment (Fig. 4D). We also noticed an accumulation of the “intact mitoSRAI” band when *Atg5* was knocked down in both hypoxic and normoxic conditions (Fig. 4C), which we suspect is because of the required role of Atg5 in promoting all autophagy events which are stalled when it is downregulated even under basal conditions (**S** Fig. 1). Confirming our prior results from the RNAi knockdown experiments, mitophagy readouts were significantly decreased in the *Atg5* null (*Atg5^5cc5^*) mutants (Fig. 4A and 4B) compared to the control group.

We next tested the impact of the knockdown of the late autophagosomal SNARE protein, STX17, on hypoxia-induced mitophagy readout. We observed significant reduction of mitophagy readout with mitoSRAI when we knocked down *Stx17* in larval muscles (Fig. 4E and 4B). Additionally, because *Stx17* knockdown permits the formation of the mitophagosome but prevents lysosomal fusion and degradation, we find what appears to be stalled mitophagic cargos that are double positive for YPet and TOLLES shown in the zoom image (Fig. 4E). To test if the small, rounded cargos double positive for YPet and TOLLES signal observed in *Stx17* knockdown were indeed stalled mitophagy cargos, we reasoned that we would observe a similar phenotype in hypoxia with pharmacologic inhibition of the lysosome. Therefore, we fed larvae exposed to hypoxia with the lysosome inhibitor chloroquine, which resulted in significantly reduced mitoSRAI mitophagy reportage and accumulation of similar stalled mitophagic cargos (showed with white arrows in zoom image) under hypoxia (Fig. 4F and 4G). Overall, these results suggest that mitoSRAI is sensitive to and specifically reports mitophagy.

### matrix-QC but not mitoSRAI is sensitive to other conditions of autophagy and mitophagy induction in vivo

**Figure 5:**
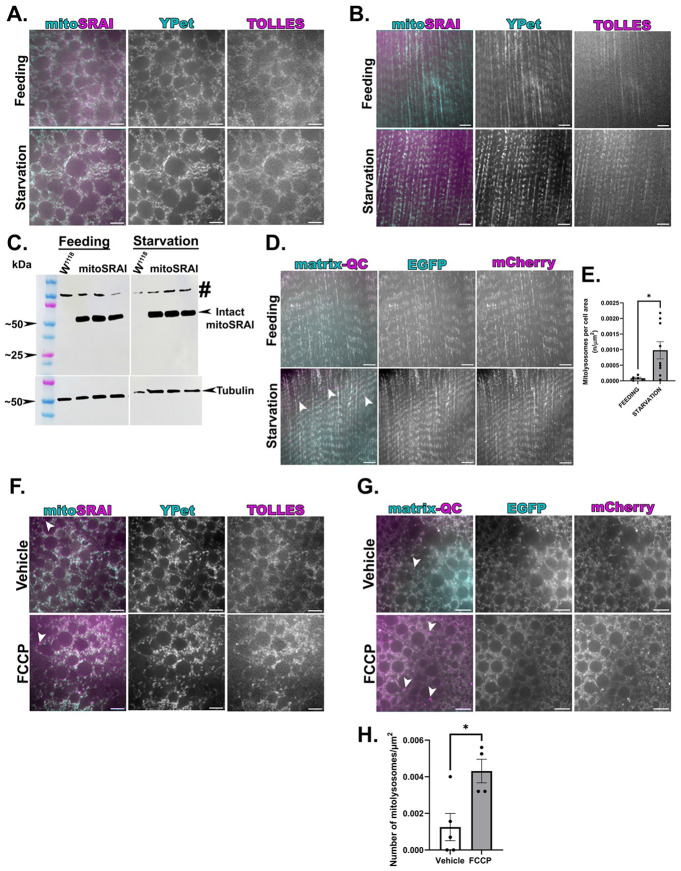
Testing mitoSRAI readout in alternative mitophagy and autophagy stimulating conditions in comparison to matrix-QC. **A**, **F**, and **G** are representative fat body images from L3 expressing mitoSRAI or matrix-QC via the cg-Gal4 driver. **B**, and **D** are representative images from L3 body wall muscles expressing mitoSRAI or matrix-QC via the Mef2-Gal4 driver. **A.** mitoSRAI expressed in the fat body in feeding and starvation conditions. **B.**mitoSRAI expressed in the larval body wall muscles in feeding and starvation conditions. **C.** Western blot image showing protein lysates obtained from the larval body wall muscles expressing mitoSRAI via the Mef2-Gal4 driver in feeding and starvation conditions. α-TOLLES antibody was used to probe for intact mitoSRAI and TOLLES only bands in each sample. # indicates nonspecific bands. **D.** Representative images of L3 body wall muscles expressing matrix-QC via Mef2-Gal4 under feeding and starvation conditions. White arrow heads are examples of mitolysosomes. Scale bar 10μm. **E.**Quantifications of mitophagy index from conditions in D. Bars represent mean, with error bars representing SEM. Individual points represent one larva with n=5 larvae in feeding condition and n=9 larvae in starvation condition, *p=0.0355. **F, G.** mitoSRAI and matrix-QC dynamics in the fat body in vehicle and FCCP treated conditions at 10 hours with white arrow heads indicating examples of mitolysosomes. Scale bar 10μm. **H.** Quantification of mitophagy index from conditions in **G**. Bars represent mean, with error bars representing SEM. Individual points represent one larva with n=5 larvae in vehicle and n=4 larvae in FCCP condition. *p<0.05.

Having shown mitoSRAI’s capability in reporting hypoxia-induced mitophagy in muscles, we explored the utility of this reagent in alternative mitophagy-inducing conditions. Starvation is a demonstrated inducer of bulk autophagy (5, 52) and has been shown to potentially include mitochondrial contents for lysosomal degradation (53). The Dm fat body is popularly used to study starvation-induced autophagy (52, 54). With this foundation, we expressed the mitoSRAI in the fat body larvae (via the cgGal4 driver) and exposed these animals to starvation. Contrary to our expectation, we did not see mitolysosomes (Fig. 5A). We confirmed autophagy induction in our system by staining for lysosomes with lysotracker red dye after starving larvae and observed increase in the number of lysosomes (**S** Fig. 2A). Consistent with previous bulk autophagy studies (26, 48, 53) and our positive results from the hypoxia-induced mitophagy in the L3 muscles, we tested the mitoSRAI readout of mitophagy in larval muscles under starvation conditions. We confirmed autophagy induction in the larval muscles by tracking the levels of the expressed tagged autophagy indicator, Atg8A-mCherry, which we found to increase in number under starvation (**S** Fig. 2B). We did not observe mitolysosomes when we starved larvae expressing mitoSRAI in the muscle (Fig. 5B), which was corroborated by the absence of “TOLLES only” bands in both fed and starved conditions on western blotting (Fig. 5C).

To compare these results with another matrix targeted mitophagy reporter (matrix-QC), we performed similar experiments with the matrix-QC reporter. When we probed starvation-induced mitophagy in larval muscles using the matrix-QC reporter, we documented mitolysosomes (Fig. 5D) which were significantly upregulated in muscle from starved animals compared to fed conditions (Fig. 5E). Not surprisingly, we witnessed high variability in starvation-induced mitophagy flux (Fig. 5E) which attests to the stochasticity of bulk autophagy events including mitochondrial components for degradation.

We next tested mitophagy readout of both reporters using a standard drug (FCCP)-inducing mitophagy assay (55). We fed fat body-expressing mitoSRAI larvae with FCCP in the treated group and DMSO (Vehicle) in the control group for 10 hours. We noticed infrequent mitolysosomes in both conditions that did not seem to differ (Fig. 5F). In contrast, when we probed the tendency of matrix-QC to report FCCP-induced mitophagy, we encountered frequent mitolysosomes (Fig. 5G) that were significantly higher in the FCCP-fed group compared to the control (vehicle) group (Fig. 5H). Similar to the results from starvation, these results suggest that matrix-QC may be more responsive to known other mitophagy-inducing conditions than mitoSRAI.

### Unlike mitoSRAI, matrix-QC is sensitive to neuronal mitophagy flux perturbations

**Figure 6:**
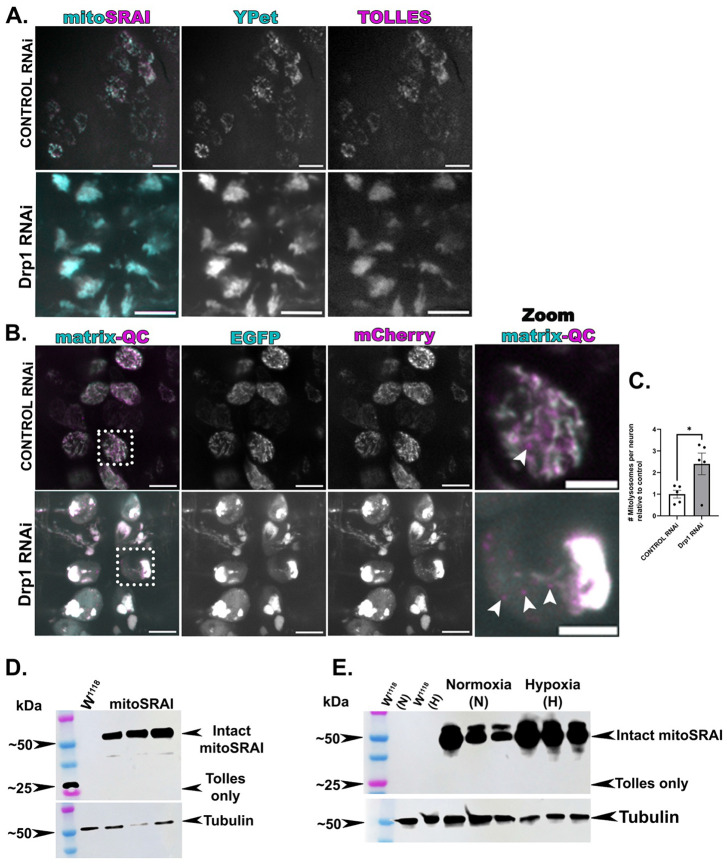
Testing neuronal mitoSRAI readout under genetic and hypoxic conditions in comparison to matrix-QC. **A.** Representative images of larval VNC co-expressing (via D42-Gal4) mitoSRAI and indicated RNAi on the left. Scale bars 10μm, and 5μm (for *Drp1* RNAi). **B.**Representative images of larval VNC (via D42-Gal4) co-expressing matrix-QC and indicated RNAi on the left. White arrow heads in zoom images indicate instances of mitolysosomes. Scale bars 10μm, 2μm (for zoom image). **C.** Graph of quantification of relative mitophagy index from conditions in B. Bars represent mean, with error bars representing SEM. Individual points represent one larva with n=5 larvae in each condition. 2-3 larval VNC images were analyzed to generate the values for the quantification. *p<0.05. **D.** Western blot image showing protein lysates obtained from the adult fly heads expressing mitoSRAI via the nSyb-Gal4 driver to probe basal neuronal mitoSRAI mitophagy readout. Each lane represents an independent sample of 15-20 fly heads. α-TOLLES antibody was used to probe for intact mitoSRAI and TOLLES only bands in each sample. **E.**Western blot image showing protein lysates obtained from the adult fly heads expressing mitoSRAI via the nSyb-Gal4 driver in normoxic and hypoxic conditions. Each lane represents an independent sample of 15-20 fly heads. α-TOLLES antibody was used to probe for intact mitoSRAI and TOLLES only bands in each sample.

Neurons are metabolically active postmitotic cells that are inextricably dependent on appropriate mitochondrial homeostasis for their survival (2, 56, 57). In agreement with this, they have been predicted to exhibit high basal (unmanipulated) mitophagy rates though a recent report of the underlying activities of some negative regulators of neuronal autophagy may suggest a low basal neuronal mitophagy rate (58). Experimentally, researchers have observed basal neuronal mitophagy endpoints using previously validated fluorescent reporters (25, 26, 41, 51). Accordingly, we tested whether we could observe basal neuronal mitophagy events in larval CNS (central nervous system) expressing (via D42-Gal4 and nSybGal4) mitoSRAI. Contrary to published literature, we did not observe basal mitophagy with mitoSRAI in control RNAi conditions (Fig. 6A). Concordantly, we did not observe basal mitolysosomal bands in western blots of lysates from adult fly brains (Fig. 6D). We have previously shown that knocking down the mitochondrial fission protein, Drp1, elevates neuronal mitophagy rates in larval motoneurons (9, 10, 59). When we reproduced this perturbation in our mitoSRAI-expressing larval CNS, we did not see an increase in mitolysosomes (Fig. 6A). To ensure our *Drp1* KD system induces mitophagy, we evaluated end-stage mitophagy with the alternative reporter matrix-QC in specific neuronal conditions of *Drp1* knock down. When we knocked down *Drp1* in larval CNS expressing matrix-QC, we observed a greater number of “mCherry only” puncta (mitolysosomes) per neuron (Fig. 6B) which was significantly different by approximately 2.5 folds relative to the control RNAi (Fig. 6C), consistent with our previously published works (9, 59). Furthermore, we found mCherry only puncta observable in basal conditions in the control matrix-QC-expressing larval CNS (Fig. 6C), an occurrence we did not observe with mitoSRAI.

There have been ambivalent reports on neuronal mitophagy response to physiological stressors like hypoxia. The most recent study on this topic observed increased neuronal mitophagy from hypoxia exposure in mice (60) while an earlier study reported inhibited mitophagy in neuronal hypoxia-ischemia condition in mice (61). With this discordant background, we sought to use our system to examine hypoxia-induced neuronal mitophagy with the mitoSRAI reporter; especially because the muscle showed elevated mitophagy in response to hypoxia (Fig. 3A and 3B). When we explored the hypoxia treatment in adult fly brain neurons expressing mitoSRAI (via western blotting), we did not observe mitophagy flux represented by the “TOLLES only” band (Fig. 6E). Our results suggest that neuronal mitophagy may be less responsive to hypoxia although we do not necessarily know how representative this is given the varying reports in the field. More studies are needed to reconcile these findings.

### BNIP3 is the principal receptor protein mediating hypoxia-induced mitophagy in Dm larval muscles

**Figure 7:**
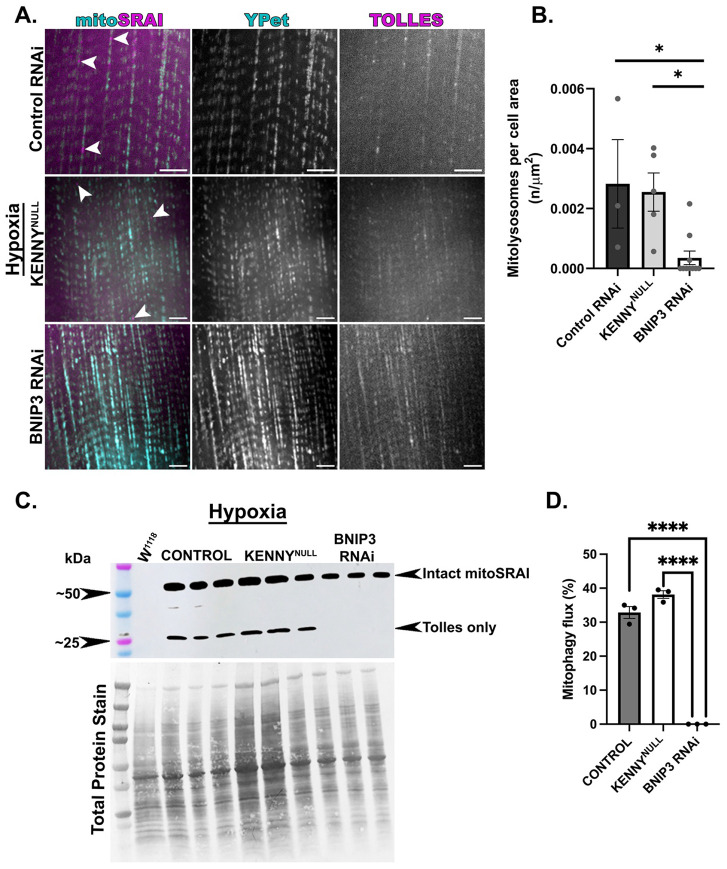
The role of BNIP3 for hypoxia-induced mitophagy in Dm. **A.** Representative images of larval body wall muscle expressing (via Mef2-Gal4) mitoSRAI with indicated genetic manipulation indicated on the left. White arrow heads pointing to magenta structures (TOLLES only) indicate examples of mitolysosomes. Scale bars 5μm (Control RNAi), 10μm. **B.** Quantification of mitophagy index from conditions in A, *p<0.05. Bars represent mean, with error bars representing SEM. Individual points represent one larva with n=3 larvae in Control RNAi, n=10 larvae in *Bnip3 RNAi*, and n=5 larvae in *Kenny^null^* conditions. **C.** Western blot image showing protein lysates obtained from the larval body wall muscles expressing mitoSRAI via the Mef2-Gal4 driver, in the genetic conditions indicated above. α-TOLLES antibody was used to probe for intact mitoSRAI and TOLLES only bands in each sample. **D.** Mitophagy flux quantification of C, ****p<0.0001. Each lane represents an independent sample of 5-6 larvae; n=3 independent samples were analyzed for each condition. Bars represent mean, with error bars representing SEM.

Hypoxia has been proposed to induce mitophagy by upregulating the mitophagy receptor, BNIP3, via the HIF1α (hypoxia-inducible factor 1α) transcription factor (50). While BNIP3 mediates a ub-independent form of mitophagy, prior work has shown contributions of both ub-dependent and ub-independent mitophagy to hypoxic stimuli (62). We have recently shown that ub-dependent mitophagy in Dm is mediated by the optineurin homolog, Kenny (9, 10). Therefore, we tested whether the loss of Kenny (ub-dependent) or downregulation of BNIP3 (ub-independent) would affect hypoxia mediated mitophagy in the muscle. We found that downregulating BNIP3 and not the loss of Kenny significantly reduced mitophagy readout under hypoxia on both microscopic (Fig. 7A and 7B) and biochemical evaluations (Fig. 7C and 7D). Notably, both the control RNAi and Kenny null group showed comparably higher mitophagy readouts than the *Bnip3* KD group (Fig. 7B and 7D). These results suggest that BNIP3 is an essential mediator of the hypoxia-induced mitophagy in the Dm larval muscles.

## Discussion

Mitophagy, a form of selective autophagy, is a fundamental process for the survival of cells and whole organisms (2, 3). Unsurprisingly, improper mitophagy regulation is associated with a devastating range of conditions like neurological diseases and cancer (56, 57). An attempt to correct these disorders via therapeutics partly revolves around elucidating the rate at which mitophagy (mitophagy flux) (23, 63) occurs in these conditions and experimentally modulating it. Studies assessing accurate ways to measure and quantify mitophagy flux in vitro and in vivo have become increasingly important to evaluate diseases stemming from mitophagy defects especially in pharmaceutical research applications. The mainstay for mitophagy detection has been electron microscopy since its first discovery (64). Although considered the gold standard, quantification of mitophagy is crude and impractical for large scale applications. Isotopic or radiolabeling of long-lived mitochondrial proteins in core autophagy (*Atg5*) mutants (53, 65, 66) and assessing their rates of breakdown have been implied as an indirect measure of mitophagy flux. This approach is susceptible to inaccuracy because changes in mitochondrial protein levels do not necessarily represent mitochondrial degradation and other forms of mitochondrial quality control may likely account for some of the mitochondrial protein changes. Moreover, labeled proteins could be degraded by autophagy-independent pathways like the ubiquitin proteasomal system. The advent of FMRs allow for relatively easy direct visualization and more accurate quantification of endpoint mitophagy and are more yielding to large scale drug discovery applications.

The prevailing challenges of the established reporters warranted the need for the progressive generation of novel FMRs, recently the mitoSRAI reporter. The mitoSRAI reporter has been well characterized in vitro with some in vivo applications (23, 67). We provide a robust in vivo validation of mitoSRAI reporter using Dm as a model in this article.

We demonstrate that mitoSRAI is mitochondrial in the initial characterization experiments in Fig. 1. Additionally, we validate the TOLLES-specific antibody (α-TOLLES) which strongly recognizes mitoSRAI on biochemical assays (Fig. 2C, 2D and 2E). This is especially helpful because it provides an alternative to possibly challenging immunohistochemistry on some tissues for more feasible western blotting assessments of mitophagy flux. mitoSRAI robustly reported mitolysosomes in larval muscles under hypoxia, a known inducer of receptor-mediated mitophagy (50). There was low basal mitophagy flux reportage in the larval muscles which align with reports elsewhere (25, 41). Moreover, we see prominent “intact mitoSRAI” bands when all autophagy is inhibited (*Atg5* RNAi) under both basal and hypoxic conditions, suggesting occurrence of low basal mitophagy that only becomes evident when bulk of the pathway is blocked (**S** Fig. 1). We suspect that these prominent bands were not observed in the *Bnip3* RNAi condition under hypoxia (Fig. 3C) reasonably because of the possibility of other active mitophagy-independent quality control pathways compensating for the knockdown of *Bnip3* in the hypoxic condition. Although the regulators of hypoxia-induced receptor mediated mitophagy are well known in other model systems (14, 17, 30, 50), they are poorly delineated in Dm. We find that BNIP3 plays a significant role in effecting this mitophagy pathway (Fig. 7A and 7C) consistent with an observation made in the zebrafish model (50). This finding was supported when we manipulated the Dm ub-dependent mitophagy adaptor gene, *Kenny* (9, 10), under hypoxia and observed unaltered mitophagy flux (Fig. 7A and 7C).

It was unexpected that we did not observe mitolysosomes with mitoSRAI when we induced mitophagy with starvation and FCCP treatment in different Dm tissues (Fig. 5). We and others have indeed observed mitolysosomes in these conditions using the matrix-QC reporter, and with mitoSRAI expression in vitro (23, 25, 26, 30). We speculate that the discordance in these results might be due to an inherent difference in the physicochemical properties of the reporters. While the TOLLES protein utilized in mitoSRAI has been purported to exhibit better fluorescence signals and relative resistance compared to mCherry (applied in matrix-QC) in simulated lysosomal environments in vitro (23), we do not necessarily know about the relative persistence of these fluorescent proteins in physiological lysosomes in vivo. In alignment with our reasoning of the disconnect between the fate of FMRs in vitro and in vivo, we suspect that perhaps mCherry can persist in the lysosome for longer and therefore can more sensitively report lower levels of mitophagy. Whereas TOLLES may not persist as long, and therefore, may only be robustly detectable when high levels of mitophagy are induced like in the larval muscle hypoxia results (Fig. 3A). Future biochemical experiments might help test and illuminate these unknowns.

We converged on investigating neuronal mitophagy with mitoSRAI to improve our understanding of the pathway and its regulators in neurons and neurological dysfunctions. We could not observe TOLLES only mitolysosomes with mitoSRAI in Dm larval motoneurons despite some mitophagy flux being reported with mitoSRAI in the mouse brain neurons which were more pronounced with neurotoxin treatments (23), and in other fluorescent reporter systems (41, 42, 68). Further, recent publications encounter mitolysosomes in neuronal and glial cultures expressing mitoSRAI in basal and neurotoxic conditions (69, 70). It is possible that the contextual difference between in vitro and in vivo environments affects the dynamics of the mitoSRAI. Also, different organismal models might process the reporter differently for currently unknown reasons; the details of which need to be explored further in subsequent studies. The mitoSRAI was unresponsive to reported mitophagy-stimulating genetic perturbations of the mitochondrial essential fission protein, Drp1 (9, 59) described earlier (Fig. 6A). matrix-QC, unlike mitoSRAI was sensitive to basal neuronal mitophagy flux and mitophagy induction when *Drp1* was knocked down (Fig. 6B). We reasonably attribute these inconsistencies among the FMRs to the context of experimental systems and model organisms, and their intrinsic properties. Of note, comparable situations of disparate FMR sensitivities and mitophagy readouts have been discussed in commentaries elsewhere (71, 72). Gaining a better understanding of the properties and limits of fluorescence signals elicited by these reporters in vivo might provide important enlightenment to our findings, and future reports.

Mitophagy has been canonized as the removal of whole mitochondria for lysosomal degradation (1). However, recent detailed studies of turnover of proteins have suggested that there may be selectivity in the degradation of mitochondrial proteins fated for mitophagy (73, 74). Consistent with this notion, additional reports have demonstrated the selective isolation of components of the mitochondria for breakdown, encompassing the term “piecemeal mitophagy” (75-77). It is largely unknown whether these selective mechanisms are functioning together with non-selective removal of whole mitochondria, and at what proportions each mechanism is occurring. If there is a high proportion of selective mitophagy of mitochondrial proteins, exogenously expressed FMRs that can read out mitophagy flux only when targeted to the lysosome would depend on their passive incorporation into cargo selected for this so-called “piecemeal mitophagy”, or the selection of reporter for degradation by mitophagy. Therefore, we speculate that the readouts of mitophagy by FMRs could be an underestimation of mitophagy activity. Overall, while FMRs give us a new tool to build on, much more research is required to gain an in-depth understanding of the full array of mitochondrial quality control mechanisms employed by different cell types.

## Conclusions

In summary, the field of fluorescence-based mitophagy reportage continues to evolve, and it is crucial that we continue exploring diverse ways to understand what these chemical biosensors are reading out. Rather than determining which reporter is superior, understanding what information these FMRs really provide will help establish their limits, and correct usage and interpretation in variable contexts. Our work together with others continues to guide and shape the application of these mitophagy-measuring tools which have large implications for translational and therapeutic discovery.

## Supplementary Material

This is a list of supplementary files associated with this preprint. Click to download.

• SuppFig.docx

## Abbreviations

ATG: Autophagy-related
BNIP3: BCL2 and adenovirus E1B 19-kDa-interacting protein 3
CCCP: Carbonyl cyanide m-chlorophenyl hydrazine
CNS: Central Nervous System
COXVIII: Cytochrome c Oxidase subunit 8
CuSO_4_: Copper (II) Sulfate
DAPI: 4’,6-diamidino-2-phenylindole
DGRC: Drosophila Genomics Resource Center
DMSO: dimethyl sulfoxide
Drp1: Dynamin-related protein 1
DTT: dithiothreitol
EGFP: Enhanced Green Fluorescent Protein
FBS: Fetal Bovine Serum
FCCP: Carbonyl cyanide p-(trifluoromethoxy) phenylhydrazone
FP: fluorescent protein
FUNDC1: FUN14 domain-containing 1
FRET: Förster resonance energy transfer; HIF1α, Hypoxia-inducible factor 1^α^
LC3: Microtubule-associated protein 1A/1B-light chain 3
LED: light-emitting diode
matrix-QC: matrix Quality Contro
mito-QC: mitochondrial Quality Control
mitoSRAI: mitochondrial Signal-Retaining Autophagy Indicator
NDP52: Nuclear Dot Protein 52 kDa
NIX/BNIP3L: Nip3-like protein X/BNIP3-like
OMM: Outer Mitochondrial Membrane
OPTN: Optineurin
p62/SQSTM1: Sequestosome 1
PBS: Phosphate Buffered Saline
PBST: PBS with Triton X-100
PDH: Pyruvate dehydrogenase
PINK1: PTEN-induced putative kinase 1
PVDF: polyvinylidene difluoride
RMCE: recombinase mediated cassette exchange
ROI: region of interest
SNARE: Soluble NSF Attachment Protein Receptors
TBST: Tris-Buffered Saline with Tween-20
TOLLES: Tolerance of Lysosomal Environments
UAS: Upstream Activating Sequence
VNC: ventral nerve cord
